# Assessment of endothelial cell function and physiological microcirculatory reserve by video microscopy using a topical acetylcholine and nitroglycerin challenge

**DOI:** 10.1186/s40635-017-0139-0

**Published:** 2017-05-18

**Authors:** Matthias Peter Hilty, Jacqueline Pichler, Bulent Ergin, Urs Hefti, Tobias Michael Merz, Can Ince, Marco Maggiorini

**Affiliations:** 1 Medical Intensive Care Unit, University Hospital of Zurich, Rämistrasse 100, 8091 Zurich, Switzerland; 2 Department of Pulmonary Medicine, Inselspital, Bern University Hospital, Bern, Switzerland; 3 Department of Intensive Care Medicine, Inselspital, Bern University Hospital, Bern, Switzerland; 4 Department of Translational Physiology, Academic Medical Center, Amsterdam, The Netherlands; 5 Swiss Sportclinic, Bern, Switzerland

**Keywords:** Microcirculation, Hemodynamic monitoring, Video microscopy, Endothelial cell function, Vasodilator, Incident dark field

## Abstract

**Background:**

Assessment of the microcirculation is a promising target for the hemodynamic management of critically ill patients. However, just as the sole reliance on macrocirculatory parameters, single static parameters of the microcirculation may not represent a sufficient guide. Our hypothesis was that by serial topical application of acetylcholine (ACH) and nitroglycerin (NG), the sublingual microcirculation can be challenged to determine its endothelial cell-dependent and smooth muscle-dependent physiological reserve capacity.

**Methods:**

In 41 healthy subjects, sublingual capillary microscopy was performed before and after topical application of ACH and NG. Total vessel density (TVD) was assessed in parallel using manual computer-assisted image analysis as well as a fully automated analysis pathway utilizing a newly developed computer algorithm. Flow velocity was assessed using space-time diagrams of the venules as well as the algorithm-based calculation of an average perfused speed indicator (APSI).

**Results:**

No change in all measured parameters was detected after sublingual topical application of ACH. Sublingual topical application of NG however led to an increase in TVD, space-time diagram-derived venular flow velocity and APSI. No difference was detected in heart rate, blood pressure, and cardiac output as measured by echocardiography, as well as in plasma nitric oxide metabolite content before and after the topical application of ACH and NG.

**Conclusions:**

In healthy subjects, the sublingual microcirculatory physiological reserve can be assessed non-invasively by topical application of nitroglycerin without affecting systemic circulation.

## Background

The primary goal of hemodynamic management in critically ill patients in shock is to ensure adequate oxygen delivery to the tissue. Currently intervention protocols are mostly targeting global oxygen delivery by optimizing intravascular blood volume, vascular tone, cardiac output, and hematocrit [1–4], but there is an increasing awareness that global hemodynamic targets may not be adequate to achieve this goal at the level of organs and cells [5]. The assessment of the microcirculation is being proposed as a more promising target for hemodynamic management, potentially enabling optimization of oxygen delivery on a local level, and possibly also of oxygen extraction [6–9].

Multiple approaches for the assessment of the microcirculation have been suggested. These range from clinical parameters such as skin mottling [10], toe temperature [11], or near-infrared spectroscopy [12] to the direct measurement of the flow of blood cells through the venules and capillaries through laser Doppler measurements [13] and more recently, capillary microscopy [14–17]. Many of these methods have been applied to the skin, sublingual mucosa, or the surface of internal organs. The demonstration of an association between the alteration of these parameters to patient mortality in conditions of critical illness such as septic shock underscores the importance of the state of the microcirculation [10, 18]. Since the introduction of capillary microscopy, several parameters have been identified that could potentially serve as treatment targets for the hemodynamic management, such as the density of the capillary network consisting of vessels with a diameter below 25 μm (total vessel density, TVD) and flow velocity in individual capillaries. However, all these parameters bear a common weakness as they are static, rather than dynamic, and so far failed to contribute to an improved outcome in clinical practice [8, 19]. We therefore propose to introduce the concept of physiological microcirculatory reserve, corresponding to the amount of transfer capacity that can be gained by maximization of microcirculatory flow (convection reserve) and, through recruitment, TVD (diffusion reserve). Acetylcholine (ACH) through a G protein-coupled mechanism leads to the expression of endothelial nitric oxide synthase to produce nitric oxide [20–22]. In contrast, nitroglycerin (NG) enzymatically releases nitric oxide [23, 24] and thus bypasses the involvement of the endothelial cells. Nitric oxide in turn leads to relaxation of the smooth muscle cells in the walls of arterioles and venules through activation of a cGMP-regulated pathway [25]. Thus, direct ligand-receptor interaction would lend insight into endothelial cell and smooth muscle function with respect to the regulation of vascular tone. Some previous attempts have been made to assess endothelial cell function using intra-arterial application of acetylcholine (ACH) and nitroglycerin (NG) [26, 27], and also through reactive hyperemia [28]. However, both of these methods are hardly applicable in a clinical setting. The topical application of ACH and NG, on the other hand, has been demonstrated on the skin of the forehead to increase blood flow velocity as measured by laser Doppler [13]. In a small number of patients in septic shock [29] and heart failure [30], the sublingual topical application of ACH reversed some of the microcirculatory alterations caused by the clinical condition as measured by sidestream dark field imaging. The aim of the present study was to expand on this previous research and derive a non-invasive method to assess physiological microcirculatory reserve. Our hypothesis was that (I) using serial topical application of ACH and NG, the microcirculation can be challenged to determine its endothelium cell-dependent and smooth muscle-dependent physiological reserve capacity by increasing TVD and microcirculatory flow velocity and that (II) topical application of ACH and NG in doses needed for local microcirculatory stimulation do not cause measurable effects in the systemic circulation. As a secondary aim, we have tested whether (III) the intervention induced changes in TVD and microcirculatory flow velocity can be detected both by manual computer-assisted video analysis representing the current gold standard, as well as a newly developed, fully automated algorithm for microcirculatory video analysis. In order to test our hypothesis, we have examined 41 healthy subjects using incident dark field video microscopy and assessed TVD and microcirculatory flow velocity before and after the topical application of ACH and NG both by manual computer-assisted video analysis and by a newly developed, fully automated algorithm.

## Methods

This study was approved by the Ethics Committee of the University of Bern (KEK 226/12, ClinicalTrials.gov identifier NCT01953198) and conducted in accordance with the declaration of Helsinki.

### Study population and design

Fourty-one healthy Caucasian persons (age 45.8 ± 1.9 years, 22/41 (54%) male, weight 69.0 ± 1.8 kg, height 174 ± 1 cm, BMI 23.1 ± 0.8 kg/m^2^) were included in the study. All were physically active but not involved in elite sport. All subjects underwent examination of the sublingual microcirculation by capillary microscopy before and after topical application of ACH and NG. According to a predetermined study time plan, clinical and echocardiography examinations as well as blood sampling were performed before the examination of the microcirculation in one group of subjects (*n* = 30), and less than 1 h afterwards in the remaining subjects (*n* = 11).

### Clinical and laboratory examination

Heart rate as well as systemic arterial blood pressure were obtained non-invasively using the oscillometric technique. Venous blood was sampled from an antecubital vein into heparinized containers and was immediately centrifuged and deep frozen on site. Plasma nitric oxide metabolite content was then measured following ethanol-induced protein precipitation and centrifugation using a vanadium(III) assay to reduce nitrate, S-nitrosothiol and nitrite to nitric oxide that was then detected in a helium driven, ozone/I_3_-based chemiluminescent nitric oxide analyzer (Sievers, Model 280 NO analyzer, Boulder, CO, USA). The method is described in more detail elsewhere [31]. Arterial blood was sampled from a radial artery after successful completion of Allen’s test. Blood gas analysis on arterial blood samples was performed on site without delay (Epoc System, Alere, Waltham, MA, USA).

### Echocardiography examination

Echocardiography examinations were performed using a portable device with tissue Doppler imaging capability (Vivid i Ultrasound System, GE Healthcare, Chicago, IL, USA). Stroke volume was measured by multiplying left ventricular outflow tract velocity time integral as recorded in the parasternal short axis by pulsed wave Doppler and left ventricular outflow tract area derived from its diameter. Cardiac output was obtained by multiplication of stroke volume and heart rate derived from the peak-to-peak distance of the outflow tract Doppler signal. All measurements were performed in triplicates and recorded digitally for offline assessment which was performed after removal of subject identifiable by a single operator. The means of the three respective measurements are reported.

### Assessment of the microcirculation

High-resolution capillary microscopy was performed using the incident dark field (IDF) technique [16] with a CytoCam handheld microscope (Braedius Medical, Huizen, The Netherlands) connected to a portable computer via a custom built camera controller. Videos were recorded to a solid state storage device at a rate of 25 frames per second and a resolution of 2208 × 1648 pixels covering 1.78 mm^2^. Each video clip was recorded at a length of 10 s. All examinations in this study were performed by a single operator with the subject in supine position and at rest. Four video clips of a random sublingual area were digitally recorded. Additionally, two video recordings were obtained after topical application of ACH and a futher two after topical application of NG in order to test endothelium-dependent and endothelium-independent physiological mirocirculatory reserve, respectively. All measured parameters are reported as the mean of obtained video clips in order to compensate for changes in microscope field of view in between measurements in compliance with the international consensus [32]; however, two instead of three post-intervention video clips for the ACH and NG measurements were acquired in order to limit total examination time. ACH was applied to the sublingual area to be analyzed as one drop (0.05 ml) of 1% (6.8·10^−2^ M) ACH solution immediately after reconstitution of ACH lyophilisate (Miochol E, Bausch & Lomb Swiss, Zug, Switzerland) using distilled water, yielding a dose of 0.5 mg (3.4 μmol) per application. Following the recording of the two ACH video sequences, NG was applied to the sublingual area to be analyzed as one drop (0.05 ml) of 1% (4.4·10^−2^ M) NG solution (Perlinganit isotonic infusion solution, UBC Pharma, Bulle, Switzerland) diluted 1:10^2^ with 0.9% sodium chloride, yielding 0.005 mg (2.2·10^−2^ μmol) per dose.

### Analysis of the microcirculatory microscopy video data

In each of the 10-s video clips recorded a stable sequence of ≥150 frames, corresponding to 6 s, was identified. The resulting video clips were graded using Massey’s scoring system [33], and analysis was performed in clips with a Massey score <10. The video files were analyzed in parallel using manual computer-assisted image analysis representing the current gold standard [34], as well as a fully automated analysis pathway utilizing a newly developed computer algorithm.

For manual video analysis, the digital video files were converted to a format and resolution compatible with previous microscopy hardware and presented in a blinded fashion. TVD was measured using software-assisted manual vessel detection (AVA 3.2; Automated Vascular Analysis, Academic Medical Center, University of Amsterdam) [34] in line with the international consensus [32] by four experienced operators that were randomly assigned to the video clips in order to prevent inter-operator bias during analysis. In order to directly characterize the intervention’s effect given the direct effect of NG on the smooth muscle cells of the venular wall, the effect of the intervention on microcirculatory flow velocity was estimated by the slope of space-time diagrams [35] generated within the center section of venules in video clips containing suitable venular anatomy. The generation of two space-time diagrams per video file was targeted; their slope was determined as the mean of three to five manually identified contrast lines representing the movement of the erythrocytes. The mean of all space-time diagrams within a video clip is reported as space-time diagram-based flow velocity.

For automated video analysis, TVD was calculated from the video files using CytoCam Tools 1.7.12 (Braedius Medical, Huizen, The Netherlands), employing an automated vessel detection algorithm based on Frangi’s multiscale vessel enhancement filtering algorithm described in detail elsewhere [36]. The algorithm was implemented using the National Library of Medicine Insight Segmentation and Registration Toolkit [37]. Vessels detected with a diameter over 25 μm were not classified as capillaries and were discarded by the software according to current guidelines [32], since larger vessels such as arterioles and venules are functionally separate from the capillary network and may be more prone to pressure artifacts. TVD was calculated by adding the lengths of all detected capillaries calibrated to a pixel size of 2.8 μm given by the microscopy hardware configuration used, divided by the area of the field of view of 1.78 mm^2^. Thereafter, a “speed image” was calculated along the centerline of all detected capillaries using a Gaussian convolution kernel, yielding not an absolute measure of speed but rather a relative speed based on intensity variation, called the speed indicator. A spatial average of the speed indicator is reported as an index of the average microcirculatory flow velocity of all perfused vessels (average perfused speed indicator (APSI)).

### Statistical analysis

Comparisons of microcirculatory parameters before and after interventions were performed using one-way analysis of variance (ANOVA). Pairwise analysis was performed using pairwise two-sample tests with Benjamini and Hochberg’s correction algorithm [38] applied. Comparisons of hemodynamic status and plasma nitric oxide metabolite content before and after interventions were performed using the Mann-Whitney *U* test. Categorical population attributes were compared using Fisher’s exact test. For correlation of TVD between manual and algorithm-based video analysis, Pearson’s product-moment correlation coefficient and Bland-Altman analysis [39, 40] were used. A two-sided *p* < 0.05 was considered statistically significant. For all statistical analysis, a fully scripted data management pathway was created within the R environment for statistical computing, version 3.3.0 [41]. Graphical output was generated using the R library ggplot2, version 2.1.0 [42]. Values are given as mean ± SEM.

## Results

### Hemodynamic status and systemic effects of the topical vasodilator application

**Fig. 1:**
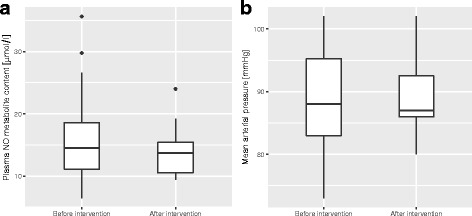
Plasma nitric oxide metabolite content (*p* = 0.66) (**a**) and mean arterial pressure (*p* = 0.48) (**b**) before and after topical vasodilator application (intervention). *Boxplots* represent median, interquartile range, and range. *NO* nitric oxide

Heart rate, systemic arterial blood pressure, and cardiac output as measured by echocardiography were within the normal range and were similar whether measured before or after the sublingual application of ACH and NG (Table 1). The groups of subjects where measurements of hemodynamic status and blood sampling were performed before and after the sublingual application of ACH and NG also had similar respiratory and metabolic status as measured by oxygen partial pressure, saturation, blood oxygen content, and lactate levels. Systemic venous plasma nitric oxide metabolite content as a direct measure of possible NG absorption via the sublingual capillaries was not increased in the blood samples taken after sublingual application of NG as compared to before (Table 1, Fig. 1).

**Table 1:** Hemodynamic status and plasma nitric oxide metabolite content before and after the sublingual topical application of acetylcholine and nitroglycerin

	Before topical application of acetylcholine and nitroglycerin (*n* = 30)	After topical application of acetylcholine and nitroglycerin (*n* = 11)	*p*
Plasma nitric oxide metabolite content [μmol/l]	15.82 ± 1.28	14.35 ± 1.59	0.66
Heart rate [1/min]	59 ± 1	61 ± 5	>0.99
Systolic arterial pressure [mmHg]	119 ± 2	126 ± 6	0.38
Mean arterial pressure [mmHg]	89 ± 2	93 ± 5	0.48
Diastolic arterial pressure [mmHg]	73 ± 2	77 ± 5	0.68
Cardiac output [l/min]	7.9 ± 0.3	7.8 ± 0.5	0.65
DO_2_ [ml/min]	1531 ± 74	1544 ± 111	0.61
SaO_2_ [kPa]	97.7 ± 0.1	97.1 ± 0.4	0.18
PaO_2_ [kPa]	12.9 ± 0.2	12.1 ± 0.5	0.21
CaO_2_ [ml O_2_/l]	193 ± 2	197 ± 3	0.24
PaCO_2_ [kPa]	5.0 ± 0.1	5.2 ± 0.1	0.24
Hb [g/l]	14.7 ± 0.2	15.1 ± 0.2	0.18
Hematocrit [%]	43.2 ± 0.5	44.4 ± 0.7	0.21
Lactate [mmol/l]	0.9 ± 0.1	0.8 ± 0.1	0.96

Values are given as mean ± SEM

*Pa* systemic arterial pressure, *DO*_*2*_ delivery of oxygen, *CaO*_*2*_ arterial oxygen content, *Hb* hemoglobin concentration

### Effects of the topical vasodilator application on the sublingual microcirculation

**Fig. 2:**
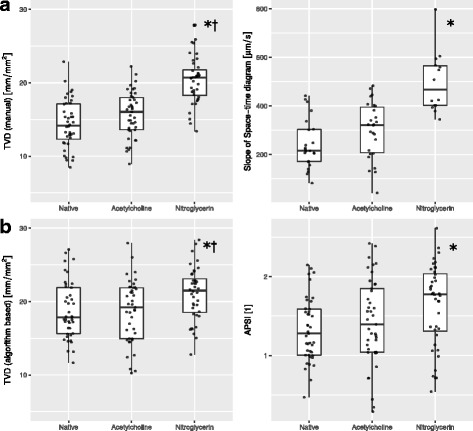
Properties of the sublingual microcirculation before (native) and after the topical sublingual application of acetylcholine and nitroglycerin. **a** Total vessel density (TVD) and space-time diagram-based flow velocity of the venules as determined using manual video analysis. ANOVA *p* < 0.0001 for TVD and *p* < 0.01 for space-time diagram-based flow velocity; asterisk denotes values differing from native examination in pairwise analysis (*p* < 0.05), dagger denotes values differing to acetylcholine stimulation in pairwise analysis (*p* < 0.05). **b** Total vessel density and average perfused speed index (APSI) as determined using algorithm-based video analysis. ANOVA *p* < 0.01 for TVD and *p* = 0.02 for APSI; asterisk denotes values differing from native examination in pairwise analysis (*p* < 0.05), dagger denotes values differing to acetylcholine stimulation in pairwise analysis (*p* < 0.05). *Boxplots* represent median, interquartile range, and range. *Horizontal scattering* is applied to the individual data points in order to avoid superimposition

**Fig. 3:**
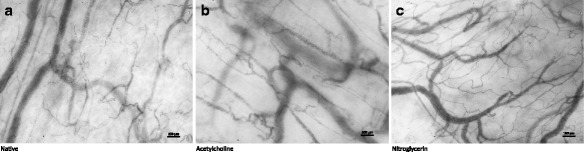
Representative still images and space-time diagrams of the venules depicting the native sublingual microcirculation (**a**) as well as after the topical sublingual application of acetylcholine (**b**) and nitroglycerin (**c**). Stimulation with nitroglycerin leads to an increase of total vessel density through recruitment of capillaries and an increase in flow velocity in the capillaries and venules

Three hundred twenty-eight video clips of the sublingual microcirculation were graded, 325 of which presented with a Massey score <10 and were provisioned to further analysis. The analyzed video clips were of good quality with a mean Massey score of 1.3 ± 0.1 (illumination 0.2 ± 0.0, duration 0.2 ± 0.0, focus 0.4 ± 0.0, content 0.3 ± 0.0, stability 0.2 ± 0.0, pressure 0.2 ± 0.0). Venular anatomy favorable for the calculation of space-time diagram-based venular flow velocity was present in 23, 27, and 14 out of 41 subjects during the native, ACH, and NG condition, respectively. Manual computer-assisted video analysis revealed no influence of topical application of ACH to the sublingual mucosa on TVD or the blood flow velocity within the venules as quantified by space-time diagrams. Topical application of NG to the sublingual mucosa however led to an increase in TVD as well as space-time diagram-based flow velocity (Table 2, Fig. 2a). Algorithm-based video analysis revealed comparable results, also demonstrating no change in TVD and APSI after the ACH intervention, and an increase in TVD as well as APSI after the NG intervention (Table 2, Fig. 2b). A moderate correlation was found between TVD as measured manually and utilizing the algorithm over all analyzed videos (*r* = 0.42, *p* < 0.001), Bland-Altman analysis revealed a bias of 2.1 mm/mm^2^ and a precision of 4.8 mm/mm^2^. Representative still images of the microcirculation before and after the application of an ACH and NG challenge are shown in Fig. 3.

**Table 2:** Properties of the microcirculation before and after the sublingual topical application of acetylcholine and nitroglycerin, as measured using manual video analysis and algorithm-based video analysis

	Native	Acetylcholine	Nitroglycerin	*p*
TVD (manual) [mm/mm^2^]	14.81 ± 0.65	15.72 ± 0.49	20.32 ± 0.50^a, b^	<0.0001
Space-time diagram-based venular flow velocity [μm/s]	242 ± 21	358 ± 62	496 ± 22^a^	<0.01
TVD (algorithm-based) [mm/mm^2^]	18.80 ± 0.61	18.69 ± 0.66	21.06 ± 0.57^a, b^	<0.01
APSI [1]	1.33 ± 0.06	1.43 ± 0.08	1.64 ± 0.08^a^	0.02

Values are given as mean ± SEM. Pairwise analysis is represented by ^a, b^ where *p* < 0.05 versus native^a^, and acetylcholine^b^. Analysis was performed in *n* = 41 subjects for TVD (manual), TVD (algorithm-based) and APSI, and in *n* = 23, *n* = 27, and *n* = 14 subjects in the native, acetylcholine, and nitroglycerin condition for space-time diagram-based venular flow velocity, respectively

*TVD* total vessel density, *APSI* average perfused speed index

## Discussion

Our study demonstrates that (I) nitroglycerin but not acetylcholine applied topically to the sublingual mucosa increases both total vessel density and local capillary flow velocity in healthy volunteers, (II) without causing measurable systemic effects. Thus, the topical sublingual application of nitroglycrine in combination with microcirculatory video microscopy provides a means to quantify the sublingual physiological microcirculatory reserve. In addition, we have employed manual computer-assisted video analysis as well as introduced an objective and reproducible algorithm for the analysis of capillary microscopy videos, (III) yielding moderately comparable results.

### Assessment of the physiological microcirculatory reserve using a nitroglycerin challenge

Previous studies have demonstrated NG to increase sublingual microvascular flow velocity in septic shock after systemic application [43–45]. The same has been demonstrated for regional perfusion after local intravascular application of NG into the arterial circulation of the arm [27] and leg [26], as well as microvascular flow velocity after transdermal application to the forehead [13]. In concordance with this data, the present study demonstrates that a relaxation of the microcirculatory smooth muscle cells can be achieved in a similar way using topical application of NG to the sublingual mucosa, leading to the measurable recruitment of both microcirculatory diffusion and convection reserve as demonstrated by the increase in TVD and microcirculatory flow velocity, respectively. Furthermore, the same effect was detected using manual video analysis, representing the current “gold standard” [32, 34], as well as employing automated algorithm-based video analysis. Potential implications of automated video analysis are the elimination of inter-observer bias, as well as representing a requirement for enabling the assessment of the microcirculation in a clinical setting.

### Assessment of the endothelium cell-dependent physiological microcirculatory reserve using an acetylcholine challenge

A previous study examining patients in cardiogenic shock has found that in a subgroup of ten patients, topical application of ACH to the sublingual mucosa led to an increase in the proportion of perfused capillaries, but no change in TVD [30]. Later, the same group described the reversal of a decrease in TVD and the proportion of perfused capillaries caused by septic shock induced by topical application of ACH in subgroup of their study [29], suggesting that local activation of the endothelial cells’ nitric oxide synthase can lead to measurable effects in the microcirculation in patients in shock. In our study, applying a similar challenge to healthy subjects does not lead to measurable changes in the microcirculation, suggesting that in healthy subjects, an ACH challenge does not add to the information obtained in unchallenged microcirculatory analysis. These results may however be influenced by the choice of local ACH application method in the present study, namely, the application of drops of ACH solution versus the placement of a tissue saturated with ACH solution as used in previous studies [29, 30] and the dose applied in the present study. The fact that Schonberger et al. have described an increase in microvascular flow velocity as measured by laser Doppler flowmetry after transdermal application of ACH to the forehead [13] may prompt the use of a different method of application or a higher dose, respectively dose-response series in future studies in healthy subjects, but foremost, it would be of interest to repeat the sublingual ACH challenge in subjects with altered microcirculation. The lack of signal in the venular space-time diagrams may further be propagated by the lack of direct effect of ACH on the smooth muscle cells within the venules’ tunica media as is expected for NG. As in both the settings of the native microcirculation as well as the NG challenge, the manual analysis and the analysis based on the automated algorithm yielded comparable results in the ACH challenge.

### Systemic effects of sublingual topical vasodilators

During topical application of 0.5 mg of ACH and 0.005 mg of NG to the sublingual mucosa in the present study, systemic arterial blood pressure, heart rate, and cardiac output as well as hematological and metabolic status remained unchanged. This corresponds to previous data reporting effects limited to the area of application after intra-ocular injection of much higher doses (5–20 mg) of the ACH preparation used in this study, Miochol E [46]. NG as used to achieve systemic effects is typically applied in sublingual application with a dose of 0.4–0.8 mg per application and in intravenous application with a dosage of 0.01–0.6 mg/min [47]. It has been previously demonstrated that systemic effects are not detected with intravenous concentrations <0.0001 mg/ml [48], and according to the relationship between sublingual and intravenous concentrations, this is equivalent to a sublingual application of 0.01 mg [49], again corresponding to our observation of the absence of a systemic effect after application of half the amount of NG in the present study. Plasma nitric oxide metabolites observed in this study before the application of NG correspond to values previously reported in patients with ischemic heart disease at rest [50] and are the same compared to after the application of NG. It is expected from previous data regarding the kinetics of nitric oxide metabolites in plasma [51, 52] that an elevation if present would have been detected within the time frame of the sample collection. The hemodynamic measurements performed after the sublingual application of nitroglycerin in this study further indicate no lasting systemic effect, even though they have to be interpreted with caution due to the variability in timing of the measurements.

### Limitations

The use of a nitroglycerin challenge to test physiological microcirculatory reserve allows the identification of the capillaries recruitable by maximal relaxation of the vascular smooth muscle cells; however, it cannot be ruled out that some unrecruited vessels remain. Further, the observation of the isolated effect of NG application is overlapped by the stepwise approach consisting of the stimulation of endogenous NO release by the application of ACH followed by the provision of excess exogenous NO by the application of NG as employed in the present study. Additionally, technical limitations regarding the assessment of the microcirculation in the present study include three main points. First, the use of a frame rate of 25 frames per second during video capture, potentially leading to the inability to identify individual erythrocytes under conditions of high flow velocity. Future hardware should aim at a higher frame rate of at least 60 frames per second in order to avoid aliasing effects in the determination of microcirculatory flow velocity when used in combination with stimulation tests such as the topical application of vasodilators. Second, only two video clips were recorded after the topical application of both ACH and NG in order to limit total examination time and minimize confounding factors such as changes in the macrocirculation over time. Nevertheless, three video clips should be recorded per measurement in future studies if the clips are not recorded at the exact same mucosal location in order to limit selection bias and fully comply with the international consensus [32]. The impact of this limitation is partly mitigated by the larger field of view per location of the IDF camera setup used in the present study as compared to the equipment used in developing current recommendations. Third, in space-time diagrams of venules consisting of a parallel flow pattern, some lateral movement of erythrocytes out of the plane of velocity measurement cannot be excluded, potentially reducing the precision of the latter measurement. Furthermore, the screening for macrocirculatory hemodynamic effects of the study intervention is supported in part by the comparison of measurements in two groups of subjects. While it is preferable to compare measurements performed in the same subjects before and after the intervention, given the results of the present study, the conclusion is valid that a relevant effect of the intervention on the macrocirculation is unlikely.

### Conclusions

Our data demonstrates that in healthy subjects, the sublingual physiological microcirculatory reserve can be assessed non-invasively by topical application of nitroglycerin without affecting systemic circulation, through the mobilization of both the microcirculatory diffusion and convection reserve. Such measurements may be conducted in critically ill patients in a future study to test the microcirculatory response to resuscitation. It remains to be seen if stimulation with acetylcholine enables the differentiation of the physiological microcirculatory reserve into endothelium cell-dependent and smooth muscle-dependent fractions in subjects with altered microcirculation. Further, automated algorithm-based analysis has been found to reasonably approximate manual analysis of microcirculatory video for assessment of the physiological microcirculatory reserve.

## Abbreviations

ACH: Acetylcholine
APSI: Average perfused speed indicator
IDF: Incident dark field
NG: Nitroglycerin
TVD: Total vessel density
